# Naxos Disease^1^

**Published:** 2005-04-01

**Authors:** Nikos Protonotarios, Adalena Tsatsopoulou

**Affiliations:** Yannis Protonotarios Medical Center, Hora Naxos, Naxos 84300, Greece

**Keywords:** Arrhythmogenic right ventricular dysplasia/cardiomyopathy, Naxos disease, Cell adhesions, Sudden death

## Abstract

Since 1995, according to the World Health Organisation’s classification of cardiomyopathies, Naxos disease has been considered as the recessive form of arrhythmogenic right ventricular dysplasia/cardiomyopathy (ARVD/C). It is a stereotype association of ARVD/C with a cutaneous phenotype, characterised by woolly hair and palmoplantar keratoderma.

## Definition

Since 1995, according to the World Health Organisation’s classification of cardiomyopathies, Naxos disease has been considered as the recessive form of arrhythmogenic right ventricular dysplasia/cardiomyopathy (ARVD/C) [1]. It is a stereotype association of ARVD/C with a cutaneous phenotype, characterised by woolly hair and palmoplantar keratoderma [2].

## Epidemiology

Naxos disease was first reported in 1986 by Protonotarios et al in patients originating from the Hellenic island of Naxos [2,3]. Apart from Naxos, cases have also been reported from other Hellenic islands, as well as from Turkey, Israel and Saudi Arabia [4]. The prevalence of the disease in Hellenic islands reaches 1:1000. A variety of Naxos disease presenting at a younger age with more pronounced left ventricular involvement has been described in families from India and Ecuador (Carvajal syndrome) [5,6].

## Molecular Genetics

Genetic studies have located two causative genes, encoding for the proteins plakoglobin and desmoplakin. Plakoglobin and desmoplakin are proteins of cell-cell adhesion.

A 2-base-pair deletion mutation of the plakoglobin gene (Pk2157del2TG) truncating the C-terminal of the protein causes Naxos disease [7]. This mutation was identified in 13 families from Greece and in one family from Turkey [4,8]. The prevalence of heterozygous carriers is up to 5% of the Naxos population (20,000 inhabitants). Heterozygotes present normal phenotype except in a small minority who show woolly hair as well as a few electrocardiographic or echocardiographic abnormalities not fulfilling the criteria for ARVC [9]. Two different mutations of the desmoplakin gene (Dsp7901del1G and DspG2375R), truncating the C-terminal of the protein, have been found to underlie a similar cardiocutaneous syndrome in families from Ecuador and Israel [10,11].

## Clinical presentation and natural history

**Figure 1:**
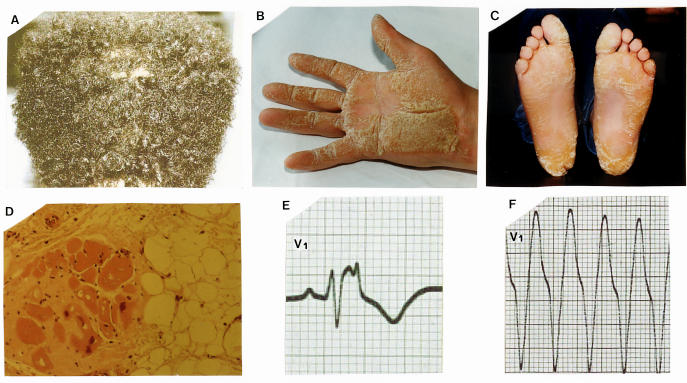
Naxos disease phenotype of the skin and heart. Woolly hair (**A**) and palmoplantar keratoderma (**B** and **C**) are the principal cutaneous abnormalities. The disease is expressed in the heart as arrhythmogenic right ventricular dysplasia. Myocardial loss and fibrofatty replacement of right ventricular myocardium (**D**) results in delayed activation (epsilon waves on surface ECG) (**E**) predisposing to reentrant ventricular arrhythmias (**F**).

In patients with Naxos disease woolly hair was apparent from birth (Figure 1A), whereas palmoplantar keratoderma developed during the first year of life as soon as the infant started to use the hands and feet (Figure 1B and C) [9,;12]. In childhood the patients were asymptomatic with no diagnostic cardiac findings. During adolescence all affected members presented electrocardiographic and/or echocardiographic abnormalities fulfilling the criteria for ARVC [13]. Resting 12-lead electrocardiogram was abnormal in more than 90% of patients [9]. Electrocardiographic abnormalities included inverted T waves in leads V1 to V3 or across the precordial leads (77%), QRS complex prolongation in leads V1 to V3 (73%), epsilon waves (42%) (Figure 1E) and complete or incomplete right bundle branch block (35%) [9]. Low voltage and/or flat T waves in left precordial leads were mostly observed in severe right or biventricular involvement. Ventricular extrasystoles of left bundle branch block configuration were recorded in the majority of patients. Ventricular extrasystoles of right bundle branch block configuration were less common. All patients presented right ventricular structural/functional alterations; minor alterations, consisting of mild dilatation or regional hypokinesia, were detected in 27% while major, consisting of severe dilatation, diffuse hypokinesia and aneurysms mostly of the outflow tract, apex or inferior wall, were detected in 73% of patients [9]. In one fourth of patients left ventricular abnormalities ranging from regional hypokinesia particularly of posterior wall or apex to diffuse dilatation and global hypokinesia were detected [9].

The symptomatic presentation was usually with syncope and/or sustained ventricular tachycardia during adolescence with a peak in young adulthood [14]. During follow-up arrhythmic events occurred in half of the patients [9]. The ECG during ventricular tachycardia always showed a left bundle branch block pattern (Figure 1F) with a frontal plane axis ranging from -30 to -60 degrees or +60 to +165 degrees [14]. The inducibility of sustained monomorphic ventricular tachycardia in the electrophysiology laboratory was high among those presenting with a clinical episode. Heart disease progressed during time to the right or both ventricles. Congestive heart failure developed in one fourth of patients at the end stage of severe right or biventricular involvement.

The annual total cardiac mortality rate was 3% and the annual sudden death mortality rate reached 2.3% [9]. Syncope, left ventricular involvement and the appearance of symptoms and/or structural progression before the age of 35 years were risk factors for sudden death [9].

Cardiac histology revealed the typical pattern of ARVD/C15 with fibrofatty replacement of right ventricular myocardium mainly in subepicardial and mediomural layers being regionally transmural with formation of aneurysms [4,16]. Surviving myocytes surrounded by fibrosis were embedded within fatty tissue (Figure 1D). Involved left ventricular myocardium showed fibrofatty or fibrous replacement [4]. Lymphocyte infiltrates were observed particularly when the biopsy was performed at the time of clinical progression. Cardiac histology in one patient from Ecuador with the Carvajal variant of Naxos disease differed only in that the fatty component of replacement process was absent [17]. Immunohistochemical studies, showed that the signal of the mutated protein (plakoglobin or desmoplakin) and the signal of connexin43 were diminished at intercellular junctions [17,18].

## Pathogenesis of ARVD/C in Naxos disease

Myocardial cells are differentiated bipolar cells coupled at intercalated discs where adherence junctions, desmosomes and gap junctions are located [19]. Adherence junctions and desmosomes secure mechanical coupling while gap junctions serve electrical coupling. Plakoglobin (γ-catenin) is the only known common component of both adherence junctions and desmosomes functioning also as a signaling molecule apart from its structural role in securing the intercellular adhesion [20]. At the adherence junctions it is connected to the actin cytoskeleton and at desmosomes to the intermediate filaments of desmin. Desmoplakin is also a cytoplasmic protein of the desmosomes that interlinks plakoglobin or plakophilin with desmin intermediate filaments. Defects in linking sites of these proteins can interrupt the contiguous chain of cell adhesion, particularly under conditions of increased mechanical stress or stretch, leading to cell isolation and death, progressive loss of myocardium and fibro-fatty replacement [4,7]. The degree of participation of fat in the repair process may be related to the rate of disease progression or may be mutation specific [4]. Surviving myocardial fibers within fibro-fatty tissue provide a slow conduction substrate inducing re-entrant ventricular arrhythmias [21]. Recent studies on Naxos disease revealed that the genetically determined defect in cell adhesion results in early gap junctions remodeling and altered electrical coupling which may act synergically with the progressive pathologic changes in myocardium contributing to a highly arrhythmogenic substrate [18].
